# A Candidate Drug Screen Strategy: The Discovery of Oroxylin A in Scutellariae Radix Against Sepsis *via* the Correlation Analysis Between Plant Metabolomics and Pharmacodynamics

**DOI:** 10.3389/fphar.2022.861105

**Published:** 2022-05-19

**Authors:** Lingyu Han, Yue Yuan, Xinyi Chen, Jian Huang, Guan Wang, Chao Zhou, Jianjian Dong, Na Zhang, Yuxin Zhang, Hang Yin, Yunyao Jiang

**Affiliations:** ^1^ School of Pharmaceutical Sciences, Institute for Chinese Materia Medica, Tsinghua University, Beijing, China; ^2^ Key Laboratory of Bioorganic Phosphorous Chemistry and Chemical Biology (Ministry of Education), School of Pharmaceutical Sciences, Beijing Advanced Innovation Center for Structural Biology, Tsinghua-Peking Center for Life Sciences, Tsinghua University, Beijing, China; ^3^ Department of Molecular Biology, Princeton University, Princeton, NJ, United States; ^4^ Beijing Huisheng Biotechnology Co., Ltd., Beijing, China; ^5^ Waters Technologies (Shanghai) Ltd., Beijing, China

**Keywords:** anti-sepsis, plant metabolomics, Scutellariae radix, oroxylin A, TLR4/NF-κB pathway

## Abstract

Sepsis is an acute systemic infectious disease with high mortality, which urgently needs more effective treatment. Scutellariae radix (SR), a commonly used traditional Chinese medicine (TCM) for clearing heat and detoxification, contains rich natural products possessing anti-inflammatory activity. In previous studies, it was found that the anti-inflammatory activities of SR extracts from different ecological conditions varied wildly. Based on this, in the present study, a screening strategy of antisepsis active components from SR based on correlation analysis between plant metabolomics and pharmacodynamics was established, and the mechanism was explored. First of all, a mass spectrum database of SR (above 240 components) was established to lay the foundation for the identification of plant metabolomics by liquid chromatography tandem mass spectrometry (LC–MS/MS). Through the correlation analysis between plant metabolomics and anti-inflammatory activity of SR from different ecology regions, 10 potential components with high correlation coefficients were preliminarily screened out. After the evaluation of anti-inflammatory activity and toxicity at the cellular level, the pharmacodynamic evaluation *in vivo* found that oroxylin A had the potentiality of antisepsis both in LPS- and CLP-induced endotoxemia mice. Network pharmacology and Western blot (WB) results indicated that oroxylin A significantly inhibited the toll-like receptor 4/nuclear factor-kappa B (TLR4/NF-κB) signaling pathway, which was further confirmed by secreted embryonic alkaline phosphatase (SEAP) assay. Moreover, the molecular docking analysis indicated that oroxylin A might competitively inhibit LPS binding to myeloid differentiation 2 (MD-2) to block the activation of TLR4. The study provided a feasible research strategy for the screening and discovery of antisepsis candidate drugs from TCM.

## 1 Introduction

Sepsis is the acute systemic infection caused by various pathogenic bacteria invading the blood circulation to produce toxins. The symptoms include shortness of breath, fever or hypothermia, recurrent chills, leukocyte change in blood, and neutrophil increase. After aggravation, the disease can develop into septic shock, disseminated intravascular coagulation (DIC), and multiple organ failure. With high mortality, sepsis is one of the most common causes of death in the intensive care unit (ICU) (Levy et al., 2012). At present, the treatment of sepsis mainly includes the use of antibiotics, hemodynamic supporting, and controlling of primary diseases (Chang and Holcomb, 2016; Dong et al., 2017; Rhodes et al., 2017). However, with the increasing number of multidrug-resistant strains, most antibiotics have become ineffective in reducing inflammatory response (Wu et al., 2017). The long-term use of antibiotics and hormones has caused great toxic and side effects, which were easy to produce flora imbalance and drug-induced diseases. Recently, immunotherapy with anti-inflammatory drugs to block severe inflammatory response in patients with sepsis has been considered to be an effective treatment.

Sepsis can be attributed to the categories of “febrile disease” and “warm toxin” in TCM, which belongs to “heat entering blood.” Huanglian Jiedu Decoction (Yu and Wang, 2018), Qingwen Baidu decoction (Xi et al., 2015), and other heat-clearing prescriptions have been commonly used in the clinic, among which SR has been a herbal medicine with high frequency. SR, the dried root of *Scutellaria baicalensis* Georgi (Labiatae), is one of the most widely used herbal medicines used in oriental medicine (called “Huang Qin” in China). It has the effects of clearing heat, purging fire, detoxification, hemostasis, and tocolysis. Clinically, it has been widely used as anti-inflammatory, antibacterial, antivirus, and anticancer drugs (Bensky et al., 1992). SR contains flavonoids, phenylethanol glycosides, alkaloids, phenols, and other components. A variety of active components have been reported to possess significant anti-inflammatory effects (Lee et al., 2005; Seo et al., 2013; Kwak et al., 2014; Koc et al., 2020; Tseng et al., 2020). Active natural products have unique biocompatibility, novel structure skeleton, and extensive pharmacological activity, which are important resources of drug discovery (Harvey, 2008; Harvey et al., 2010; Harvey et al., 2015). It is suggested that SR can be used as a natural small-molecule compound library for screening antisepsis drugs.

SR mainly grows in northern China. The main production areas include Hebei, Inner Mongolia, Shanxi, Shandong, Shaanxi, and Gansu. Different growth environments, planting methods, cultivation years, and harvest time directly affect the synthesis and accumulation of secondary metabolites. The preliminary study found that there were significant differences in the anti-inflammatory level of SR grown in different ecological environments. Accordingly, the correlation analysis between the plant metabolomics and the anti-inflammatory activity of different ecological SR was carried out, and active components with high correlation coefficients were selected for verification and further screening. Finally, it was found that oroxylin A showed good antisepsis potential. Furthermore, the antisepsis pathway of oroxylin A was predicted by network pharmacology, which was verified using the WB and SEAP assay that oroxylin A significantly inhibited the TLR4/NF-κB pathway. In addition, it was indicated that oroxylin A might bind to the TLR4-MD2 complex to regulate the TLR4/NF-κB pathway by molecular docking analysis. In this study, a screening strategy of antisepsis active components based on correlation analysis between plant metabolomics and pharmacodynamics was established, and the mechanism was partly explained. Our results provided meaningful support for the development of antisepsis drugs.

## 2 Material and Methods

### 2.1 Chemicals and Reagents

Baicalin (No.: YR-20022601), baicalein (No.: YR-H0079), wogonin (No.: YR-19012501), chrysin (No.: AF801102), pinocembrin (No.: AF911161), scutellarin (No.: RFS-Y01201905020), oroxylin A (No.: YR-200803), 8,8″-bibaicalein (No.: YR-CFS201901), baicalein-7-O-β-D-glucoside (No.: AF8102891), and isoacteoside (No.: YR-8032807) were purchased from Baoji Earay Bio-Tech Co., Ltd (Shanxi, China). Acteoside (No.: HA141444298) and examethasone (No.: HA060807) were bought from Baoji Herbest Bio-Tech Co., Ltd. (Shanxi, China). Dexamethasone sodium phosphate injection (No.: H12020515) was purchased from Tianjin KingYork Group Co., Ltd. (Tianjin, China). LC/MS-grade methanol and acetonitrile, and formic acid (No.: 202,674) were supplied by Fisher Scientific (Fairlawn, NJ, United States). Ultrapure water was purified using a Milli-Q Water Purification System (Merck Millipore, Germany). All other reagents were of HPLC grade or higher. Tween-20 (No.: DH358-4) was purchased from Beijing Dingguo Changsheng Biotechnology Co., Ltd. (Beijing, China).

### 2.2 Plant Metabolomics Analysis

#### 2.2.1 Plant Materials and Sample Preparation

In total, 20 batches of SR were provided by Henan Tailong Pharmaceutical Co., Ltd. in corresponding producing areas (Supplementary Table S1). All the samples were authenticated by Junli Chen (distinguished researcher of the Institute for Chinese Materia Medica of Tsinghua University). The medicinal materials of SR were powdered and passed through 65-mesh sieves. An aliquot of 10 mg of the powder (*n* = 8) was added into 10 ml of 70% methanol (v/v) with 1 µg of dexamethasone as the internal standard. The mixture was treated by ultrasonication for 15 min (40 kHz, 490 W) and then centrifuged at 13,000 rpm for 10 min. An appropriate amount of supernatant was taken and diluted with water for three times. An aliquot of 1 µl was injected for analysis. Each sample was taken appropriately and mixed in equal proportions as the quality control (QC) sample.

#### 2.2.2 LC–MS/MS Conditions

Plant metabolomics analysis was performed using a Synapt G_2_ ultra-performance liquid chromatography–quadrupole–time-of-flight mass spectrometry (UPLC-Q-TOF-MS; Waters Corporation, Milford, MA) system. The chromatographic separation conditions are shown later. A CORTECS UPLC C18 (2.1 mm × 100 mm, 1.6 μm) was used at 30°C with the flow rate maintained at 0.3 ml/min. The mobile phase (A) was acetonitrile and methanol (3:1, v/v), and mobile phase (B) was water containing formic acid (0.1%, v/v). A gradient elution program was used: 0–10 min with 5–20% A; 10–24 min with 20–30% A; 24–27 min with 30–36% A; 27–40 min with 36–75% A; and 40–42 min with 75–95% A. The UPLC-Q-TOF/MS acquisition was used in the MS mode. The MS analysis was performed in positive- and negative-ion modes equipped with an electrospray ionization (ESI) source. Leucine-enkephalin (ESI^+^, m/z 556.2771; ESI^−^, m/z 554.2615) was used as a standard for quality determination and lock mass solution. The following final MS conditions were used in the resolution mode: capillary voltage, 2.0 kV (−/+); source temperature, 100°C; desolvation temperature, 450°C; cone gas flow, 50 L/h; desolvation gas flow, 800 L/h; scan range: *m/z* 50-1200; and scan time: 0.2 s.

#### 2.2.3 Multivariate Statistical Analysis and Identification Analysis

In the analysis, five blank samples were used to balance the chromatographic column, followed by three QC samples. After each of the 10 samples, the QC samples were inserted for monitoring data acquisition performance throughout the analysis. Progenesis QI data analysis software (Waters, Wilmslow, United Kingdom) was used to process LC–MS raw data. Peak deconvolution, alignment, extraction, and normalization produced a list of mass-to-charge ratio and retention time pairs with peak intensities. The QC sample data were analyzed to ensure the reliability of the sample data. In excel table, the signal-to-noise (S/N) ratio was calculated through the QC median/blank, and the data with S/N ≥ 3 as the subsequent analysis data were filtered. The inter batch effect was normalized by the QC average of each collection batch.

The missing value and zero in corrected data within 1–40 min were replaced by 0.000000. Simultaneously, features with zero standard deviation were eliminated. For further normalization, each feature was divided by the maximum value in the features. Two modes (positive and negative) of data were combined into a dataframe, which was imported into R 4.1.0 to calculate the principal component analysis (PCA), partial least squares discrimination analysis (PLS-DA; ropls 1.24.0 package) and then figures were generated using the ggplot 2 3.3.5 package. PCA was used to identify outliers and trends, and PLS-DA was used to observe the classifications for different groups.

QC data in positive and negative modes were searched in the mass spectrum database of SR established using UNIFI software (version 1.9.2). The identification results matched with the data processed by Progenesis QI software in excel (retention time (RT) error 0.2 s, m/z error 0.005Da) were used as the data related to anti-inflammatory activity in the next step.

### 2.3 Cell Culture

Raw264.7 cells were obtained from the American Type Culture Collection (ATCC; Manassas, VA, United States) and were cultured in PRIM-1640 (Thermo Fisher Scientific, MA, United States) containing 10% heat-inactivated fetal bovine serum (FBS; Corning, MA, United States) and 1% penicillin-streptomycin (PS). To evaluate the effect of oroxylin A on the TLR4/NF-κB signaling pathway, HEK-Blue cells were transfected with TLR4 and NF-κB SEAP reporter (Yang et al., 2021). The cells were cultured in Dulbecco’s modified Eagle medium (DMEM; Gibco, CA, United States) supplemented with 10% (v/v) FBS, 50 U/ml penicillin, and 50 mg/ml streptomycin. HEK-Blue hTLR4 cells were maintained with the intervention of 100 μg/ml Normocin (InvivoGen, No. ant-nr1) and 1× HEK-Blue Selection (InvivoGen, No. hb-sel). HEK-Blue null cells (TLR4 free) were grown with 100 μg/ml Normocin and 100 μg/ml Zeocin in the media. All cells were incubated in an incubator with a humidified atmosphere of 5% CO_2_ at 37°C.

### 2.4 Cell Viability

MTT assay was used to determine the cell viability. Raw264.7 cells (1 × 10^5^ cells/well) were seeded in the 96-well plates. After incubation for 18 h, the cells were exposed to the medium along with samples at different concentrations for 24 h. Then, the medium was removed carefully, and the cells were incubated with 0.05% MTT (Solarbio Technology, Beijing, China) in a FBS-free medium at 37°C for 4 h. The supernatant was removed, and 200 μl DMSO was added before the measurement using a microplate reader at a wavelength of 490 nm. The cellular viability was normalized with the control group. All experiments were performed in triplicate.

### 2.5 Nitric Oxide (NO) Release Assay

The Griess reaction test was used to determine the nitric oxide release. Raw264.7 cells (1 × 10^5^ cells/well) were seeded in the 96-well plates, pretreated with different extracts of SR from different ecologies or corresponding compounds for 30 min, and then were treated with or without 1 µg/ml lipopolysaccharides (LPS) (from *Escherichia coli* O111:B4, L2630, Sigma, United States) for 24 h. A measure of 1% sulfanilamide in 5% phosphoric acid and 0.1% N-(1-naphthyl) ethylenediamine dihydrochloride in water (1:1) were mixed as a Griess reagent. A volume of 100 μl of Griess reagent was mixed with 100 μl of cell culture supernatant. The optical density was measured using a microplate reader at a wavelength of 540 nm. The NO inhibition rate was normalized with the LPS group. All experiments were performed in triplicate.

### 2.6 Correlation Analysis Between Chemical Components and Anti-Inflammatory Activity

The average content of each compound in each group was taken as a variable. Meanwhile, the activity data of each group were used as a variable. Pearson correlation analysis was performed on the two variables to obtain the correlation coefficient using the R language program (R 4.1.0). The positive correlation coefficient indicated that the content of this compound was directly proportional to the corresponding activity data. In addition, the linear relationship of content between groups was consistent with that of activity data. The negative correlation coefficient indicated that the content of this compound was inversely proportional to the corresponding activity, and the linear relationship was consistent.

### 2.7 Survival Assay in Mice

#### 2.7.1 Animals

For animal experiment, 6-week-old male C57BL/6 mice (20.1 ± 1.9 g) were purchased from Beijing Vital River Laboratory Animal Technology CO., Ltd. (license: SCXK 2016-0006, Beijing, China). All animals were housed in isolated ventilated cages (maxima six mice per cage) under a specific pathogen-free (SPF) controlled environment (temperature was set at 12-h light/dark cycle, 21.0 ± 2.0°C with a relative humidity of 45.0 ± 10.0%) with free access to diet and water. All animal experiments were approved by the Experimental Animal Center of Tsinghua University.

Two types of septic animal models were used in the present study. First, the appropriate dosage was determined using the LPS model, and the animal level efficacy of chrysin and oroxylin A was compared. Then, the efficacy of compounds with better effect on the LPS model was further confirmed using the CLP model with the comparison of positive drug (dexamethasone).

#### 2.7.2 Survival Assay in LPS Model Mice

For survival assay, 6-week-old male C57BL/6 mice (20.0 ± 1.0 g) were randomly divided into 4 groups according to their weight (Bate and Clark, 2014). The mice intravenously injected with solvent (2% DMSO and 10% Tween 80 in normal saline) or LPS (30 mg/kg) from *Escherichia coli* O111: B4 (Sigma, United States) were in the control and model groups, respectively. The solvent or chrysin/oroxylin A (3.0 mg/kg) with a final concentration of 0.4 mg/ml was injected 30 min before LPS treatment and administrated to mice everyday (totally 4 days). For example, a 20 g mouse needed 150 μl of intravenous injection. The number of survival and the body weight of living mice were recorded per 24 h.

#### 2.7.3 Survival Assay in CLP Model Mice

A model of polymicrobial sepsis known as cecal ligation and puncture (CLP) was utilized for further confirmation of pharmacodynamics. After the acclimatization of 3 days, male 7 weeks old C57BL/6 mice (21.0 ± 2.0 g) were randomly and equally divided into four groups: control, CLP model, oroxylin A treatment, and dexamethasone treatment (3.0 mg/kg, iv). Oroxylin A and dexamethasone were injected into mice 30 min before surgery, respectively. In order to reduce subjective factors, the person who made CLP models did not know the situation of drug administration. The mice were anesthetized by inhalation with isoflurane, and a longitudinal skin midline incision was made to expose the cecum out of the enterocoelia. The junction of the ileum and cecum was ligated with surgical sutures without disrupting the intestinal continuity. The ligated cecum was punctured with a 21-gauge needle two times to achieve sepsis conditions. Mice from the control group underwent the same surgery but without cecal ligation and puncture. The drug was administrated daily (total three times). The number of survival and the body weight of living mice were recorded per 24 h.

### 2.8 Plasma Cytokine Quantification

The plasma samples were collected by cardiac puncture after treatment for 24 h. Mouse plasma was taken out at −80°C storage environment and mixed by the vortex. The levels of cytokines IL-6, IL-1β, and TNF-α in plasma were determined using the enzyme-linked immunosorbent assay (ELISA) following the manufacturer’s instructions of ELISA kits (No. 550534, BD OptEIATM, United States).

### 2.9 Pathological Section Examination

The spleen, kidney, and lung tissues collected after treatment for 24 h were fixed in 10% neutral buffered formalin for 24 h and then embedded into paraffin blocks and sliced into 5-μm-thickness sections. The sections were baked at 65°C and then stained with hematoxylin and eosin (H&E). The sections were examined using a digital pathological section scanner (3DHISTECH Pannoramic SCAN, A18000062).

### 2.10 Target Prediction and Pathway Enrichment

The compound structure of oroxylin A was imported into the PharmMapper server (http://lilab.ecust.edu.cn/pharmmapper/index.php), SwissTargetPrediction (http://www.swisstargetprediction.ch/), and Drugbank to retrieve the associated targets of oroxylin A. The targets after the deletion of duplicates were the candidate targets of oroxylin A. The related targets of sepsis were obtained from OMIM (Online Mendelian Inheritance in Man; http://www.omim.org). The related targets were screened in OMIM with sepsis and septicemia as the keyword, respectively, and the targets after the deletion of duplicates were the related targets of sepsis.

KEGG pathway analysis was performed using the Database for Annotation, Visualization, and Integrated Discovery (DAVID, https://david.ncifcrf.gov, v6.8). The protein–protein interaction (PPI) networks of oroxylin A targets and sepsis-related targets were merged, and the intersection was uploaded. The pathways with significant changes (FDR <0.05) were identified.

### 2.11 Western Blot Assay

An appropriate amount of spleen tissue (about 50 mg) was lysed in radio immunoprecipitation assay (RIPA) lysis buffer (Bestbio, Shanghai, China) containing a blended protease inhibitor, then the supernatant was taken after centrifugation to obtain the total protein. The protein concentrations of the samples were measured using a BCA assay kit (76K00101, Dingguo Changsheng Biotechnology, Beijing, China). The equal amounts of protein (40 μg each sample) were electrophoresed on a 10% sodium dodecyl sulfate-polyacrylamide gel electrophoresis (SDS-PAGE; Beyotime Biotechnology, Shanghai, China) and electroblotted onto a 0.45-μm polyvinylidene fluoride (PVDF) transfer membranes (Millipore, Billerica, United States). After blocking with a protein blocking solution (KeyGen BioTECH, Jiangsu, China), the PVDF membranes were incubated with the following primary antibodies (Beyotime Biotechnology, Shanghai, China): myeloid differentiation primary response gene 88 (MyD88; 1:1000), phosphorylation-transforming growth factor beta-activated kinase 1 (p-TAK1; 1:1000), interleukin 1 receptor-associated kinase 4 (IRAK4; 1:1,000), phosphorylation-inhibitor of nuclear factor kappa-B kinase β (p-IKKβ; 1:1000), phosphorylation-NF-kappa B inhibitor alpha (p-IKBα; 1:1000), and p-p65 (1:1,000) at 4°C overnight. The membranes were washed, and then incubated with horse radish peroxidase (HRP) labeled goat anti-rabbit IgG (1:5000) for 1 h at room temperature. The protein expression was quantified using the gel imaging system (iBright1500, Thermo Fisher Scientific, MA, United States) after visualizing with enhanced chemiluminescence blotting detection reagents (Bio-Rad, California, CA, United States). The intensity of each was normalized by β-actin (internal control).

### 2.12 SEAP Assay

A total of 10,000 cells per well were plated in 96-well plates. The treatment group was grown in a medium supplemented with 50 μM oroxylin A. The TLR4 pathway was selectively activated by 200 ng/ml LPS both in HEK-Blue hTLR4 cells and HEK-Blue null cells. The cells were incubated overnight at 37°C in a humidified atmosphere of 5% CO_2_. The cell supernatant and Quanti-Blue (InvivoGen, No. Rep-qbs3), in accordance with the manufacturer’s recommendations, were mixed in a ratio of 1:1, and then incubated at 37°C for 2 h. Then, the SEAP signal was quantified by measuring the absorbance at 620 nm. Data were normalized with blank control. Each data point represents the average and standard deviation of at least three biological replicates.

After being induced by Quanti-Blue, the cell culture medium was replaced by a fresh medium containing 10% MTT and incubated at 37°C for 4 h. The supernatant was removed, and residual crystallization was dissolved with 100 μl DMSO. The absorbance of each well was measured using a microplate spectrophotometer at a wavelength of 490 nm. Viability was normalized with blank control and expressed as %.

### 2.13 Molecular Docking Simulation

Oroxylin A was docked against human MD-2 (hMD-2, PDB: 2E56) within Schrödinger Suite 2017-4 (Schrödinger, 2017). The structure of the protein was prepared by the default setting using the Protein Preparation Wizard, and the structure of the small molecule (oroxylin A) was constructed within Maestro and further prepared using the Ligprep program (Sastry et al., 2013; Harder et al., 2016). Then, molecular docking was performed using the Glide program by the extra-precision docking (Glide XP) method (Friesner et al., 2006). The top-scored and reasonable pose of the ligand was chosen for further analysis. All the molecular graphics were performed using PyMOL and BioRender.

### 2.14 Statistical Analysis

All data represent the mean and standard error values of three independent experiments and are presented as mean ± standard deviation (SD). The statistical comparisons were analyzed via GraphPad Prism 8.0 software. We calculated the *t*-test or one-way analysis of variance (ANOVA). *p-value under 0.05 and **p-value under 0.01 were thought to be statistically significant.

## 3 Results

### 3.1 Correlation Analysis Between Chemical Components and Anti-Inflammatory Activity of SR

**FIGURE 1:**
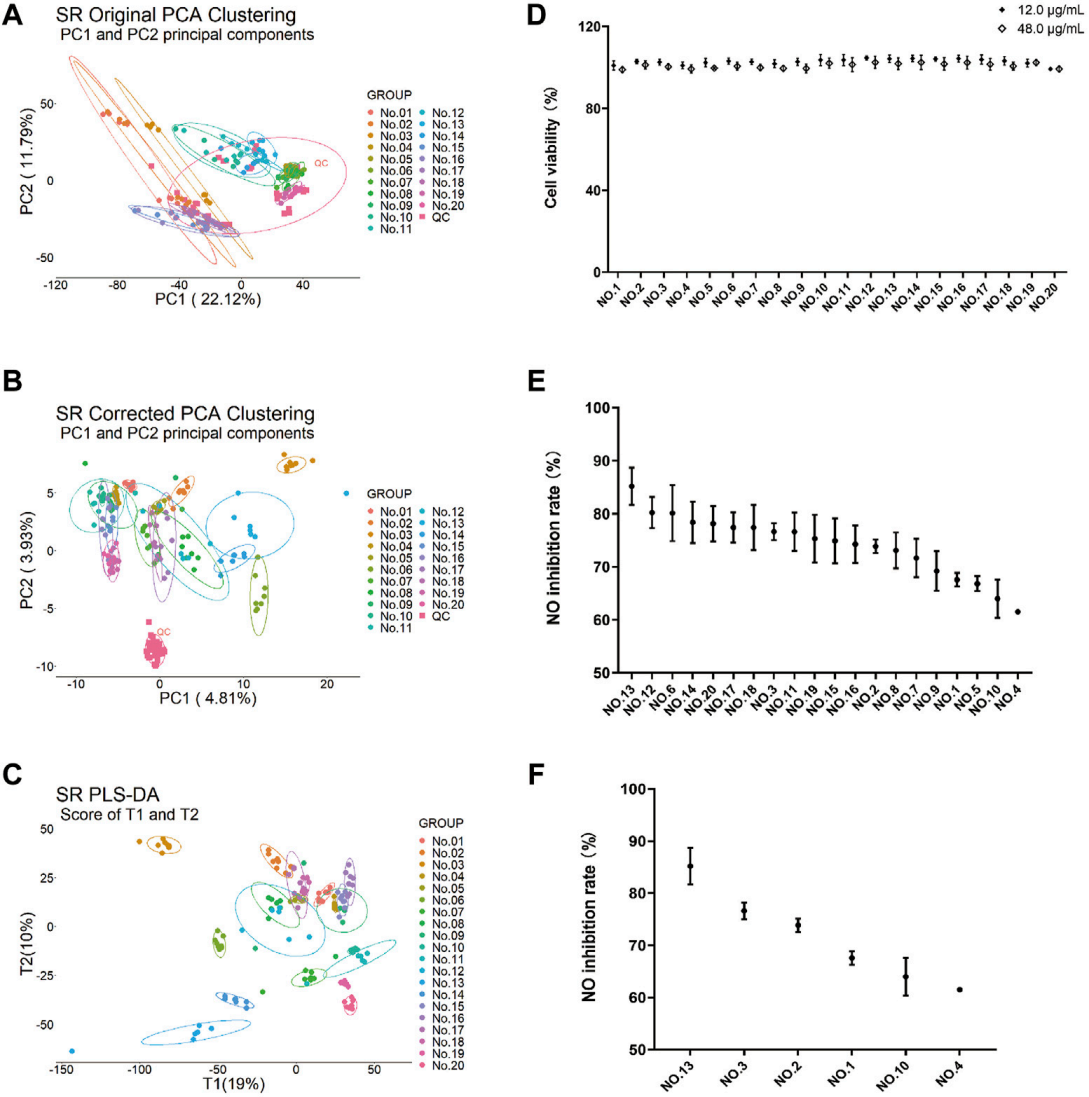
Plant metabolomics analysis and anti-inflammatory activity of 20 batches of SR extracts. PCA of 70% methanol extracts from 20 batches (*n* = 8) of SR before correction **(A)** and after correction **(B)**. **(C)** PLS-DA analysis of 70% methanol extracts from 20 batches of SR after correction. **(D)** Viability of Raw264.7 cells with 20 batches of SR extracts at 12 μg/ml and 48 μg/ml. Each value is expressed as mean ± SD (*n* = 3). **(E)** Inhibitory effects of 70% methanol extracts from 20 batches of SR at 48 μg/ml on 1 µg/ml LPS-induced NO production in Raw264.7 cells. Each value is expressed as mean ± SD (*n* = 3). **(F)** Inhibitory effects of 70% methanol extracts of 6 batches of SR with significant differences at 48 μg/ml on LPS induced NO production in Raw264.7 cells. Each value is expressed as mean ± SD (*n* = 3). Values are significantly different by Duncan’s multiple range test (*p* < 0.05).

In order to fully understand the components of SR, a mass spectrum database of SR (above 240 components) was established using UNIFI software according to the previous literature (Murch et al., 2004; Liu et al., 2009; Wang et al., 2013; Xie et al., 2015; Qiao et al., 2016). Having considered the representativeness of medicinal material samples, 20 batches of SR samples (Supplementary Table S1) at various growth years were collected from the main production areas. The plant metabolomics study of SR (70% methanol extracts) was conducted by the LC–MS/MS technology. The QC samples and other experimental samples were analyzed by unsupervised principal component analysis (PCA). In the PCA results obtained from the original data (Figure 1A), QC was distributed discretely with the mass spectrometry collection batch, indicating the influence of batch effect. After correction (Figure 1B), the relatively clustered QC samples showed that the batch effect has been eliminated. The results of corrected PCA showed that there were significant differences among different ecological SR groups. PLS-DA results (Figure 1C) also showed significant differences between groups, which further verified the results of PCA.

The data with S/N ≥ 3 were screened for qualitative analysis. After removing the duplicate value and fragments, 246 components were identified in the positive ion mode and 181 in the negative ion mode (data not provided). It laid a foundation for subsequent screening and further qualitative analysis of active components.

At the same time, the anti-inflammatory activity and cytotoxicity of SR extracts with different ecologies were also evaluated in Raw264.7 cells. In total, 20 batches of SR extracts showed no cytotoxicity at the concentration of 12 μg/ml and 48 μg/ml (Figure 1D). At the concentration of 48 μg/ml, 20 batches of SR extracts showed quite different inhibitory effects on 1 µg/ml LPS-induced NO production in Raw264.7 cells (Figure 1E). After Duncan’s multiple range test statistical analysis, the results showed that there were significant differences in the inflammatory inhibition rates of Nos. 13, 3, 2, 1, 10, and 4, which were mainly proportional to the growth years (Figure 1F).

**FIGURE 2:**
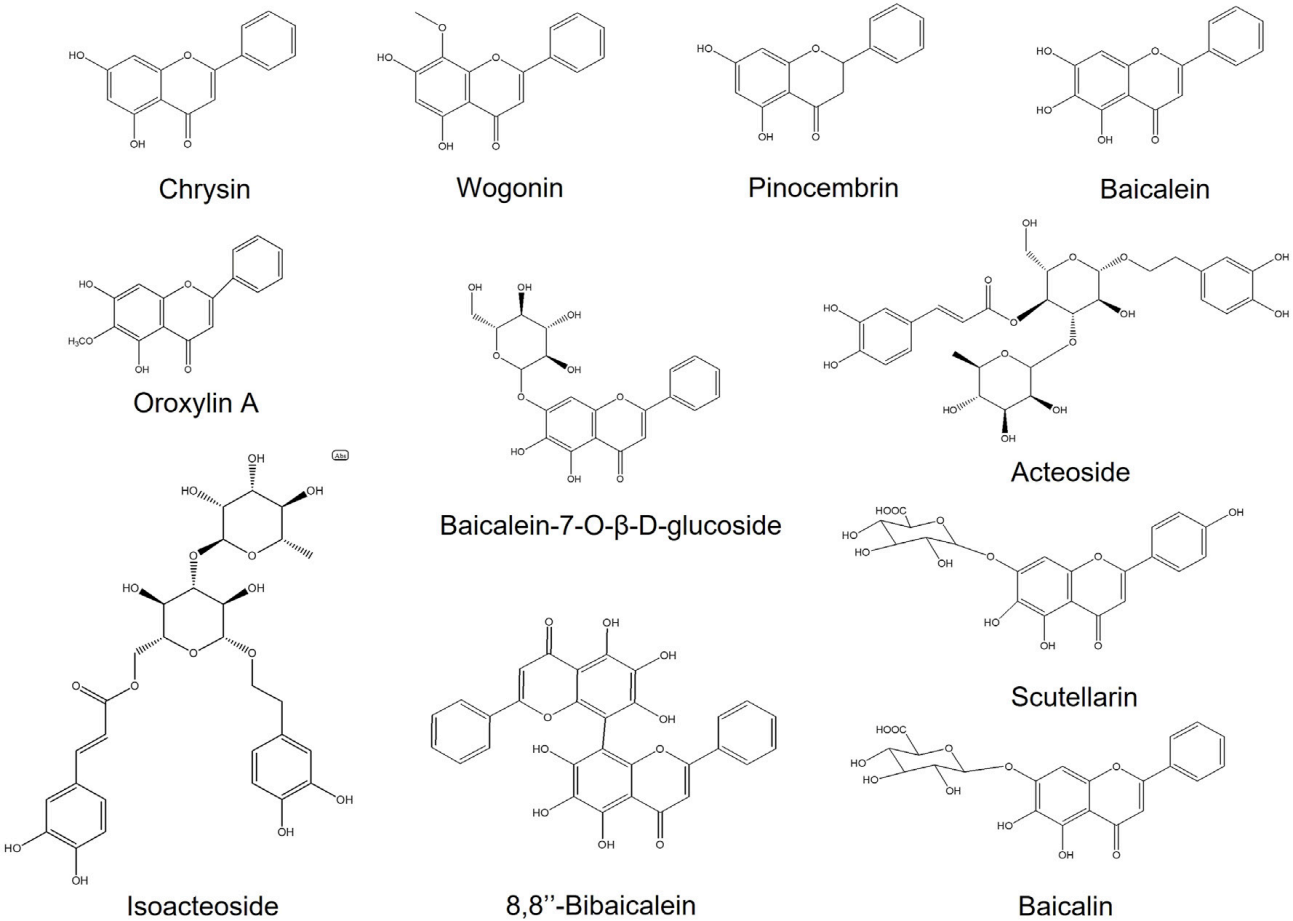
Chemical structures of components with correlation coefficient ≥80 in the positive or negative mode.

Pearson correlation analysis was used to analyze the correlation between plant metabolomics and pharmacodynamics of six batches (Nos. 13, 3, 2, 1, 10, and 4) of SR extracts. Through the SR mass spectrum database retrieval, the components with a correlation coefficient ≥80 in negative (46 compounds) and positive (53 compounds) mode were tentatively identified for further analysis (Tables 1, 2). After comparison with the reference substances, a total of 10 components (Supplementary Table S2, Figure 2) were accurately identified. Considering the high content of baicalin in SR, the subsequent activity verification of 11 components including baicalin was carried out.

**TABLE 1:** Identification of components with score ≥80 in correlation analysis of plant metabolomics and pharmacodynamics (negative mode).

No	Ion	Compound	Correlation coefficient	Identification	Formula	Molecular weight
1	neg	31.11_253.0505	96.7	**Chrysin**	C15H10O4	254.0578
2	neg	20.33_491.1187	96.6	5,2′,6′-Trihydroxy-6,7-dimethoxyflavone 2′-O-β-D-glucoside	C23H24O12	492.126
3	neg	11.09_593.1509	96.3	6-C-(β-arabinofuranosyl)- 8-C-(β-d-glucopyranosyl)-Chrysin	C26H28O13	548.1527
4	neg	10.59_431.0978	96	Baicalein-7-O-glucoside isomer	C21H20O10	432.1051
5	neg	26.23_269.0456	95.9	**Baicalein**	C15H10O5	270.0529
6	neg	31.25_255.0654	95.8	**Pinocembrin**	C15H12O4	256.0727
7	neg	13.14_477.1037	95.2	Baicalein-7-O-glucoside isomer	C21H20O10	432.1055
8	neg	18.57_431.0979	94.6	Baicalein-7-O-glucoside isomer	C21H20O10	432.1052
9	neg	17.11_581.1881	93.6	2′,4′,6′-Trihydroxydihydrochalcone-3′-C-β-D-glucoside-6′-O-β-D-glucoside	C27H34O14	582.1954
10	neg	17.44_301.0712	93.6	Pinocembrin isomer	C15H12O4	256.073
11	neg	14.29_477.1036	93	Baicalein-7-O-glucoside isomer	C21H20O10	432.1054
12	neg	30.68_283.0614	92.7	**Wogonin**	C16H12O5	284.0687
13	neg	9.75_563.1404	92.1	Schaftoside isomer	C26H28O14	564.1477
14	neg	17.64_271.0611	91.8	Dihydronorwogonin	C15H12O5	272.0684
15	neg	11.72_623.198	91.6	**Acteoside**	C29H36O15	624.2053
16	neg	26.18_283.061	90.6	Oroxylin A isomer	C16H12O5	284.0683
17	neg	12.97_431.0977	90.3	Baicalein-7-O-glucoside isomer	C21H20O10	432.105
18	neg	16.56_431.0985	90.2	**Baicalein-7-O-β-D-glucoside**	C21H20O10	432.1057
19	neg	13.21_621.1457	89.9	Wogonin-glc-o-glucuronide	C28H30O16	622.1529
20	neg	13.37_637.1405	89.9	Trihydroxy-methoxyflavone-O-glc-gluA	C28H30O17	638.1477
21	neg	14.35_637.2136	89.4	Leucosceptoside A	C30H38O15	638.2208
22	neg	20.63_651.2287	89.3	Cistanoside D isomer	C31H40O15	652.2359
23	neg	17.64_447.0935	89.2	Dihydrobaicalin	C21H20O11	448.1008
24	neg	12.83_623.198	88.5	**Isoacteoside**	C29H36O15	624.2053
25	neg	31.59_283.061	88.4	**Oroxylin A**	C16H12O5	284.0683
26	neg	6.41_475.182	88.3	Daredroside B	C21H32O12	476.1893
27	neg	16.23_637.2135	88.3	2-(3-Hydroxy-4-methoxyphenyl)-ethyl-1-O-α-L-rhamnosyl (1→3)-β-(4- O -feruolyl)glucoside	C30H38O15	638.2207
28	neg	21.46_491.1187	88.2	5,2′,6′-Trihydroxy-6,7-dimethoxyflavone 2′-O-β-D-glucoside	C23H24O12	492.126
29	neg	12.82_445.1137	88.1	Oroxylin A-7-O-β-D-glucoside isomer	C22H22O10	446.121
30	neg	15.34_637.2133	88.1	2-(3-Hydroxy-4-methoxyphenyl)-ethyl-1-O-α-L-rhamnosyl (1→3)-β-(4- O -feruolyl)glucoside	C30H38O15	638.2206
31	neg	18.78_651.2291	88	Cistanoside D	C31H40O15	652.2363
32	neg	9.92_637.1406	87.5	Trihydroxy-methoxyflavone-O-glc--gluA	C28H30O17	638.1479
33	neg	18.58_475.1244	87	8-Methoxy-5-O-glucoside flavone	C22H22O9	430.1262
34	neg	3.61_461.1659	86.8	Darendroside A	C20H30O12	462.1732
35	neg	20.46_301.0707	86.8	7,2′,6′-Trihydroxy-5-methoxychalcone	C16H14O6	302.078
36	neg	20.45_621.1446	85.8	Wogonin-glc-o-glucuronide	C28H30O16	622.1519
37	neg	9.44_581.1873	85.4	2′,4′,6′-Trihydroxydihydrochalcone-3′-c-β-D-glucoside-6′-O-β-D-glucoside	C27H34O14	582.1946
38	neg	15.05_505.098	84.8	Viscidulin II-2′-O-β-D-glucuronide	C23H22O13	506.1053
39	neg	25.57_299.0558	83.6	Daidzein	C15H10O4	254.0576
40	neg	10.28_463.0882	83.5	(2S)-5,7,2′,5′-Tetrahydroxyflavanone 7-O-β-D-glucuronopyranoside	C21H20O12	464.0954
41	neg	12.98_593.1504	82.4	6-C-(β-arabinofuranosyl)- 8-C-(β-d-glucopyranosyl)-Chrysin	C26H28O13	548.1522
42	neg	11.01_447.0926	81.9	Dihydrobaicalin	C21H20O11	448.0998
43	neg	11.45_463.0879	81.6	(2S)-5,7,2′,5′-Tetrahydroxyflavanone 7-O-β-D-glucuronopyranoside	C21H20O12	464.0952
44	neg	11.03_461.0723	81.2	**Scutellarin**	C21H18O12	462.0796
45	neg	24.98_329.0661	80.4	Raderianin	C17H14O7	330.0733
46	neg	24.52_283.0606	80.1	Oroxylin A isomer	C16H12O5	284.0678

Note: Bold, identified by comparing with reference standards.

**TABLE 2:** Identification of components with score ≥80 in correlation analysis of plant metabolomics and pharmacodynamics (positive mode).

No.	Ion	Compound	Correlation coefficient	Identification	Formula	Molecular weight
1	pos	21.94_433.1127	98.2	Baicalein-7-O-glucoside isomer	C21H20O10	432.1054
2	pos	22.11_433.1125	98.2	Baicalein-7-O-glucoside isomer	C21H20O10	432.1052
3	pos	31.14_255.0654	96.5	**Chrysin**	C15H10O4	254.0582
4	pos	13.43_477.1027	96.4	Hispidulin-7-O-glucuronide	C22H20O12	476.0954
5	pos	12.87_270.0521	96.2	Norwogonin isomer	C15H10O5	270.0527
6	pos	30.71_285.0762	94.9	**Wogonin**	C16H12O5	284.0689
7	pos	31.28_257.08	94.5	**Pinocembrin**	C15H12O4	256.0727
8	pos	7.48_417.1179	94.2	Chrysin-8-C-β-D-glucoside	C21H20O9	416.1106
9	pos	12.99_447.0919	93.9	Norwogonin-7-O-glucuronide isomer	C21H18O11	446.0847
10	pos	11.13_433.1129	93.6	Baicalein-7-O-glucoside isomer	C21H20O10	432.1056
11	pos	20.5_623.1596	93.5	Wogonin-glc-o-glucuronide	C28H30O16	622.1523
12	pos	13.41_463.1227	93.4	(2S)-7,2′-Dihydroxy-5-methoxyflavanone-7-O-β-D-glucuronopyranoside	C22H22O11	462.1155
13	pos	17.4_303.0868	93.2	7,2′,6′-Trihydroxy-5-methoxychalcone	C16H14O6	302.0795
14	pos	26.3_255.065	93.2	Daidzein	C15H10O4	254.0577
15	pos	11.04_417.1177	92.9	Chrysin-8-C-β-D-glucoside	C21H20O9	416.1104
16	pos	26.29_271.0608	92.9	**Baicalein**	C15H10O5	270.0536
17	pos	9.95_639.1553	92.5	Trihydroxy-methoxyflavone-O-glc-gluA	C28H30O17	638.148
18	pos	14.39_501.0998	92.5	5,7,2′-Trihydroxy-6-methoxyflavonone 7-O-β-D-glucuronide	C22H22O12	478.1106
19	pos	30.71_891.1592	92.5	Unkown	C42H34O22	890.1519
20	pos	9.8_565.155	92	Schaftoside isomer	C26H28O14	564.1478
21	pos	26.3_323.0529	91.7	5,7,2′-Trihydroxy-6′-methoxyflavone	C16H12O6	300.0637
22	pos	10.62_271.06	91.6	Norwogonin isomer	C15H10O5	270.0528
23	pos	26.3_319.0815	91.6	(2S)-5,7,2′,5′-Tetrahydroxy-6-methoxyflavanone	C16H14O7	318.0742
24	pos	14.39_303.0865	91.2	7,2′,6′-Trihydroxy-5-methoxychalcone	C16H14O6	302.0792
25	pos	12.27_309.0755_2	91	Dihydrooroxylin A	C16H14O5	286.0863
26	pos	31.61_285.0760	90.5	**Oroxylin A**	C16H12O5	284.0687
27	pos	16.6_433.1131	89.7	**Baicalein-7-O-β-D-glucoside**	C21H20O10	432.1058
28	pos	11.77_647.1938	89.3	**Isoacteoside**	C29H36O15	624.2045
29	pos	12.98_623.1235	89.1	Baicalein-6,7-di-O-glucuronide	C27H26O17	622.1162
30	pos	18.74_303.0867	89	7,2′,6′-Trihydroxy-5-methoxychalcone	C16H14O6	302.0794
31	pos	13.03_433.1127	88.3	Baicalein-7-O-glucoside isomer	C21H20O10	432.1054
32	pos	20.66_675.2252	88.2	Cistanoside D isomer	C31H40O15	652.2359
33	pos	11.12_303.0502	87.8	3,6,7,2′,6′-pentahydroxyflavone	C15H10O7	302.0429
34	pos	13.61_549.1599	87.7	6-C-(β-arabinofuranosyl)- 8-C-(β-d-glucopyranosyl)-chrysin	C26H28O13	548.1526
35	pos	31.99_539.0970	87.4	**8,8′′-Bibaicalein**	C30H18O10	538.0897
36	pos	17.73_431.097	87.1	5,7-Dihydroxy-6,8-dimethoxyflavone	C17H14O6	314.079
37	pos	18.81_485.1652	86.2	Darendroside A	C20H30O12	462.176
38	pos	12.89_647.1938	85.9	**Isoacteoside**	C29H36O15	624.2046
39	pos	21.51_447.1286	85.8	Oroxylin A-7-O-β-D-glucoside	C22H22O10	446.1213
40	pos	18.65_433.1128	85.5	Baicalein-7-O-glucoside isomer	C21H20O10	432.1055
41	pos	13.27_270.0526	85	Norwogonin isomer	C15H10O5	270.0531
42	pos	18.81_675.2255	85	Cistanoside D	C31H40O15	652.2363
43	pos	10.41_411.1075	84.9	5-Hydroxy 7,8,2′,5′,6′-Pentamethoxyflavone	C20H20O8	388.1183
44	pos	19.81_417.1179	84.8	Chrysin-8-C-β-D-glucoside	C21H20O9	416.1107
45	pos	14.41_661.2091	84.5	2-(3-Hydroxy-4-methoxyphenyl)-ethyl-1-O-α-L-rhamnosyl (1→3)-β-(4- O -feruolyl)glucoside	C30H38O15	638.2199
46	pos	6.44_499.1779	84.2	Daredroside B	C21H32O12	476.1887
47	pos	15.14_507.1129	84.2	Viscidulin II-2′-O-β-D-glucuronide	C23H22O13	506.1056
48	pos	13.27_623.1598	83.1	Wogonin-glc-o-glucuronide	C28H30O16	622.1525
49	pos	14.91_463.1229	82.7	(2S)-7,2′-Dihydroxy-5-methoxyflavanone 7-O-β-D-glucuronopyranoside	C22H22O11	462.1156
50	pos	25.35_301.071	81.7	5,7,2′-Trihydroxy-6′-methoxyflavone	C16H12O6	300.0638
51	pos	25.63_301.0709	81.7	5,7,2′-Trihydroxy-6′-methoxyflavone	C16H12O6	300.0636
52	pos	27.79_623.1602	80.7	Wogonin-glc-o-glucuronide	C28H30O16	622.1529
53	pos	12.27_411.1059_2	80.6	5-Hydroxy 7,8,2′,5′,6′-Pentamethoxyflavone	C20H20O8	388.1167

Note: Bold, identified by comparing with reference standards.

### 3.2 Verification and Screening of Anti-Inflammatory Activity of Active Components in Raw264.7 Cells

**FIGURE 3:**
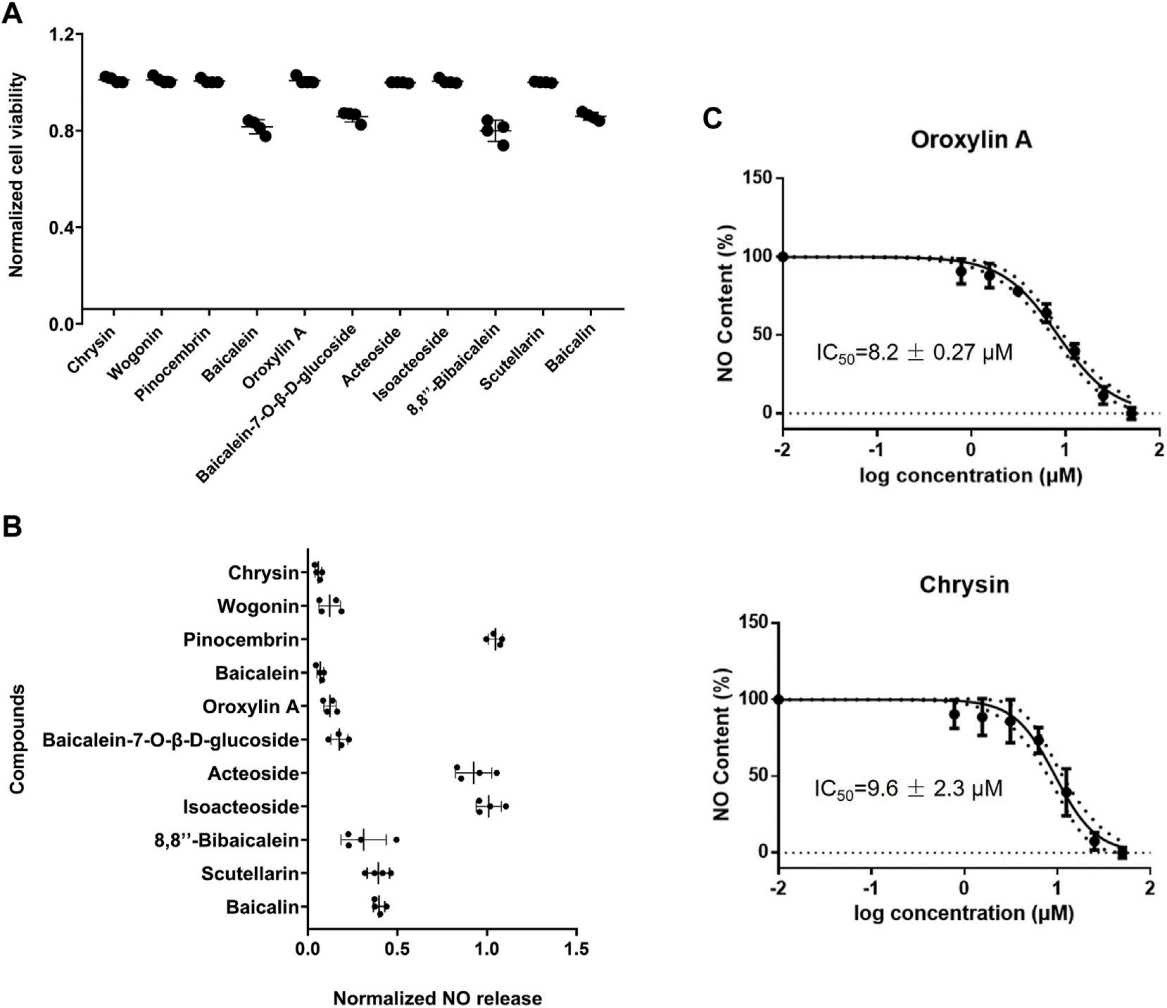
Verification and screening of anti-inflammatory activity of active components in Raw264.7 cells. **(A)** Viability of Raw264.7 cells. Cells were treated with 50 μM corresponding reagents and then measured by MTT assay, following by the normalization of cell viability. **(B)** Screening results in Raw264.7 cells. Cells were treated with 1 µg/ml LPS and 50 μM corresponding reagents, and then measured the release of NO, followed by the normalization of inhibition rate. **(C)** Dose-dependent inhibitory response of LPS-induced NO release in Raw264.7 cells. Cells were treated with 1 µg/ml LPS and serial concentration of chrysin and oroxylin A (0.78, 1.56, 3.12, 6.25, 12.5, 25, and 50 μM) and then measured the release of NO, followed by the normalization of inhibition rate. All data were reported as the mean of three independent experiments.

The cell level anti-inflammatory activity and toxicity of the aforementioned 11 potential anti-inflammatory active components were measured in this section. Baicalein, baicalein-7-O-β-D-glucoside, 8,8″-bibaicalein, and baicalin showed cytotoxicity at the concentration of 50 μM (Figure 3A). At the concentration of 50 μM, pinocembrin, acteoside, and isoacteoside showed poor anti-inflammatory activity in Raw264.7 cells by detecting nitric oxide (NO) release (Figure 3B). Wogonin, baicalein, and scutellarin showed strong cytotoxicity at the concentration of 100 μM (Supplementary Figure S1A). According to the results of anti-inflammatory activity and cytotoxicity, it was found that chrysin and oroxylin A displayed superior activity and lower toxicity among the 11 compounds. The IC_50_ values of chrysin and oroxylin A were 9.6 ± 2.3 μM and 8.2 ± 0.27 μM, respectively (Figure 3C). In addition, the results of MTT showed that within 50 μM these two compounds processed no cytotoxicity (Supplementary Figures S1B,C).

### 3.3 Pharmacodynamic Evaluation of Antisepsis Effect in Mice

#### 3.3.1 Oroxylin A Significantly Improving the Survival Rate of Endotoxemia Mice

**FIGURE 4:**
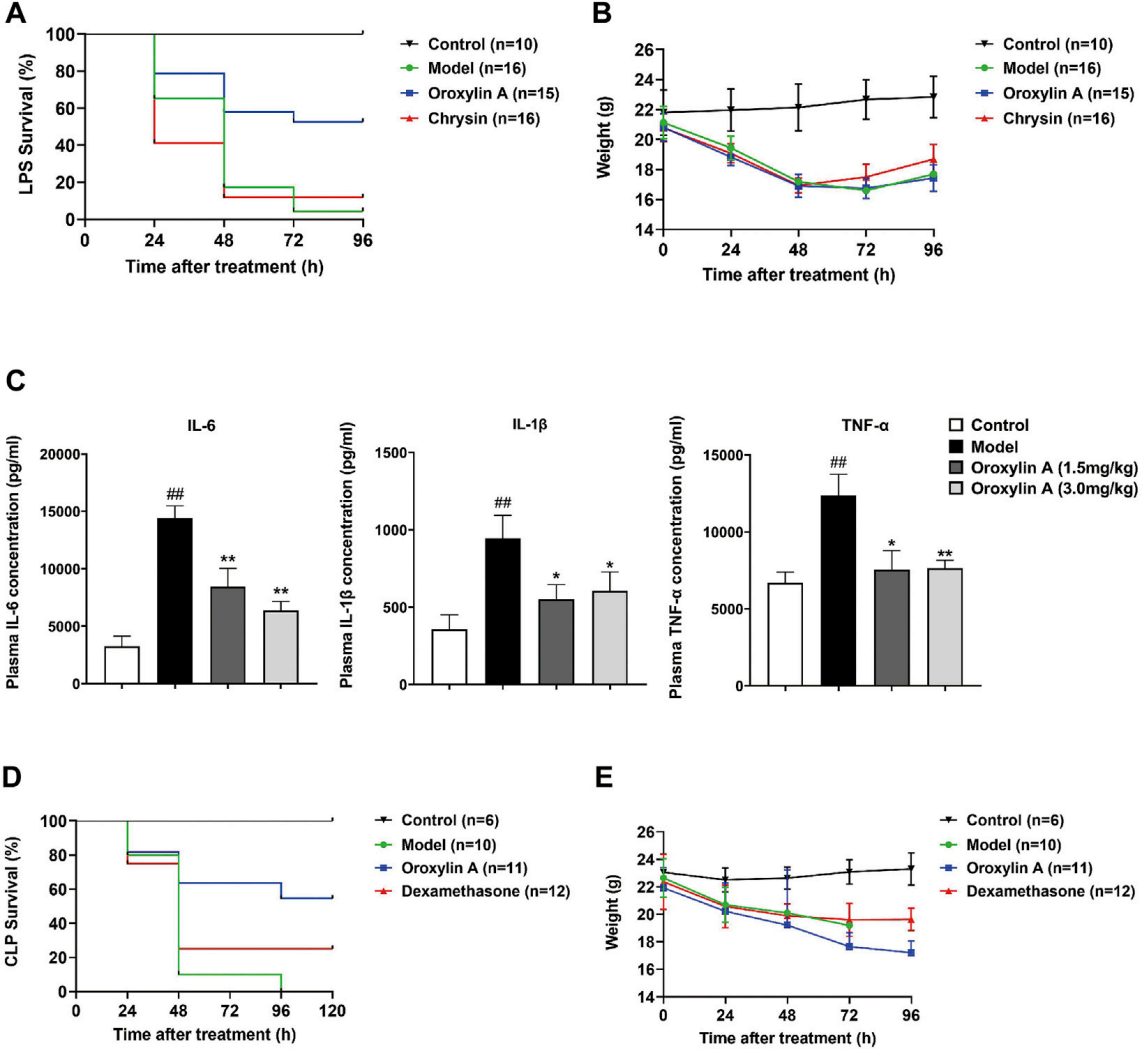
Protection of oroxylin A on endotoxemia mice. **(A)** Survival rates of LPS-induced endotoxemia mice with administration of oroxylin A and chrysin. **(B)** Body weight of LPS-induced endotoxemia mice with administration of oroxylin A and chrysin. **(C)** Inhibitory response of pro-inflammatory cytokine IL-6, IL-1β, and TNF-α in LPS-induced endotoxemia mice plasma by different doses of oroxylin A (*n* = 3). Oroxylin A and chrysin (3.0 mg/kg) were injected to mice every day after LPS challenge (30 mg/kg, iv). The first-time treatment was 30 min prior to the injection of LPS. **(D)** Survival rates of CLP-induced endotoxemia mice with administration of oroxylin A and dexamethasone. **(E)** Body weight of CLP-induced endotoxemia mice with administration of oroxylin A and dexamethasone. Oroxylin A and dexamethasone (3.0 mg/kg) were injected 30 min before mice subjected to CLP. Drugs were administrated every day. Data are presented as mean ± SD. ##*p* < 0.01 vs. control, *p < 0.05 vs. model, **p < 0.01 vs. model.

In order to further compare the therapeutic effects of chrysin and oroxylin A, LPS was injected into male C57BL/6J mice to induce endotoxemia. As shown in Figure 4A, the survival rate of endotoxemia mice with oroxylin A intervention was about 45%, which was significantly higher than that of LPS group (5%). The survival rate of endotoxemia mice administrated with chrysin was about 10%, slightly higher than that of LPS group. The body weight of surviving mice was significantly reduced after LPS induction, and then began to recover after 72 h (Figure 4B). In addition, the compounds at the dose of 3.0 mg/kg were nontoxic (treated daily) within 96 h through the body weight monitoring (Supplementary Figure S1D).

Since oroxylin A significantly improved the survival rate of endotoxemia mice, its anti-inflammatory ability was further evaluated. The secretion of pro-inflammatory cytokines (IL-1β, IL-6 and TNF-α) in the plasma after being treated for 24 h was detected (Figure 4C). It can be seen that oroxylin A significantly reduced the expression of pro-inflammatory cytokines, suggesting that oroxylin A could effectively suppress systemic inflammation in endotoxemic mice. However, the anti-inflammatory effect did not show significant concentration dependence at the concentrations of 1.5 mg/kg and 3.0 mg/kg.

To further verify the therapeutic effect and to determine whether the effect of 0roxylin A was limited to the induction of LPS, a CLP model inducing polymicrobial sepsis was established (Hubbard et al., 2005). The survival rate of endotoxemia mice treated with 3.0 mg/kg of oroxylin A was about 50%, significantly higher than that of positive control (dexamethasone) and the model group (Figure 4D). The body weight continued to decline within 96 h after oroxylin A treatment, but the descent rate slowed down after 72 h (Figure 4E). The survival mice were in good condition during the 1 week follow-up observation, proved that oroxylin A has good antisepsis potential.

#### 3.3.2 Oroxylin A Protecting Organs With Severe Inflammation in Endotoxemic Mice

To evaluate the organ protection effect of oroxylin A on LPS-induced endotoxemic mice, the pathological sections of the spleen, lung, and kidney after treatment for 24 h were analyzed as follows.

**FIGURE 5:**
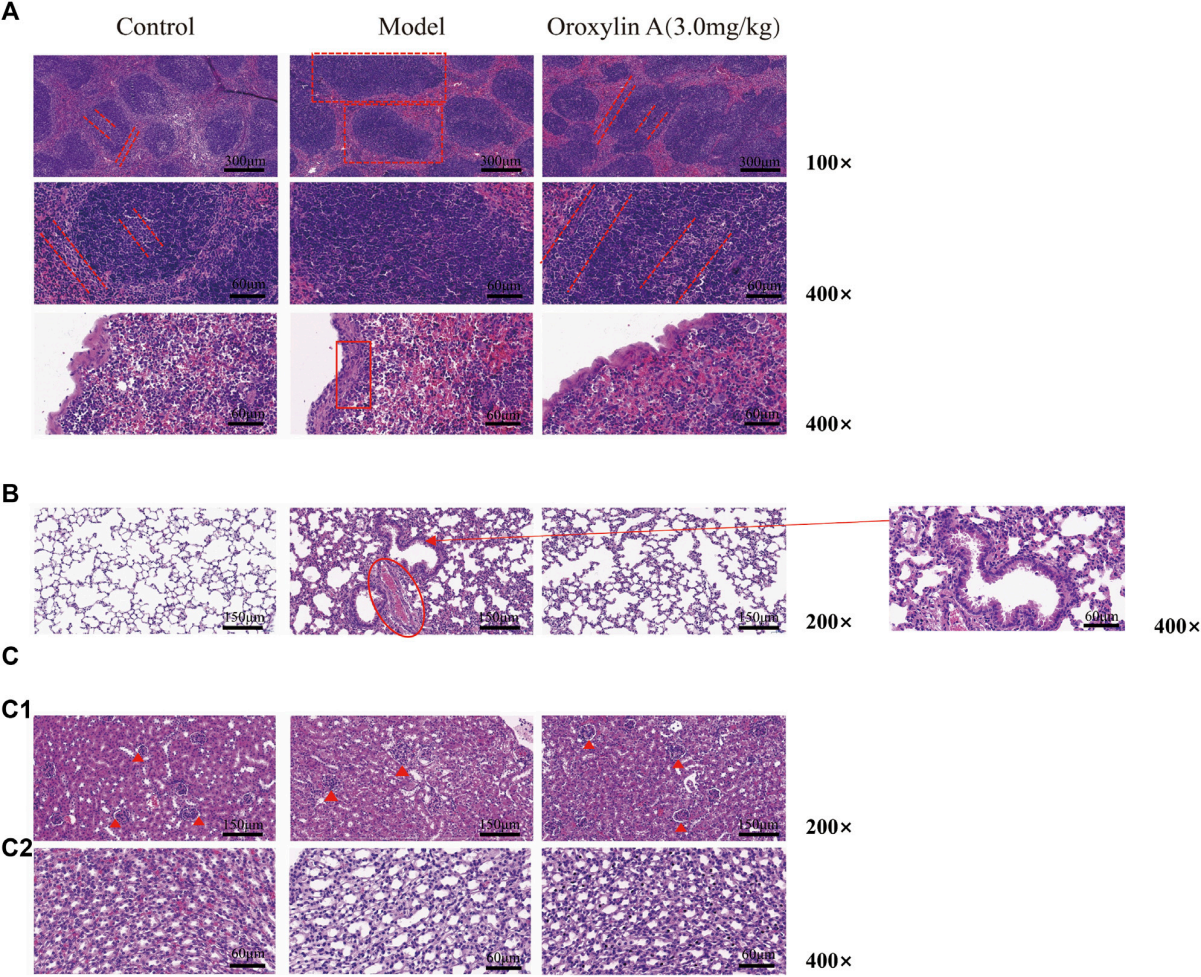
Organ protection of oroxylin A on LPS-induced endotoxemia mice. H&E-stained spleen **(A)**, lung **(B)**, and kidney **(C)** sections of control, model, and treatment groups. Oroxylin A was 30 min prior to the injection of LPS (30 mg/kg, iv). Tissues were collected after treatment for 24 h. The number of mice in each group is greater than or equal to three.

In Figure 5A, it can be seen that the white pulp nodule was swollen and structurally disorganized with the germinal center and marginal zone disappeared (dotted panes) after LPS induction. Moreover, serious inflammatory cell infiltration in the velamen of the spleen (panes) suggested serious inflammatory changes in the spleen. After oroxylin A intervention, although the germinal center and marginal zone were slightly blurred compared to the control, the white pulp nodule was obviously restored (dotted parallel lines) with improved swelling and significantly reduced inflammatory cell infiltration in velamen. Figure 5B shows the pathological changes in the lungs. The alveoli in the control group were clear and complete, and the mucosal cilia were in order. In the model group, thickening of alveolar wall, congestion between alveoli (rings), lodging of cilia in alveolar mucosa (arrows), and partial alveolar edema were observed. After being treated with oroxylin A, alveolar walls, congestion, and mucociliary lodging were improved. The pathological changes in the kidney were shown in Figure 5C. Compared to the control, the glomerular lumen became thin and adhered to the glomerulus (solid triangles) in the LPS treated group (Figure 5C1 **)**. The swollen renal tubule lumen and some degenerative epithelial cells were also noticed (Figure 5C2 **)**. After being treated with oroxylin A, the glomerular sac almost returned to the state of the control group. Meanwhile, the lumen swelling and morphology of epithelial cells were improved to some extent. These results indicated that oroxylin A could effectively reduce organ inflammation and injury in endotoxemic mice.

### 3.4 Mechanism Research of Oroxylin A on Sepsis

#### 3.4.1 Target Prediction and Pathway Prediction of Oroxylin A on Sepsis

**FIGURE 6:**
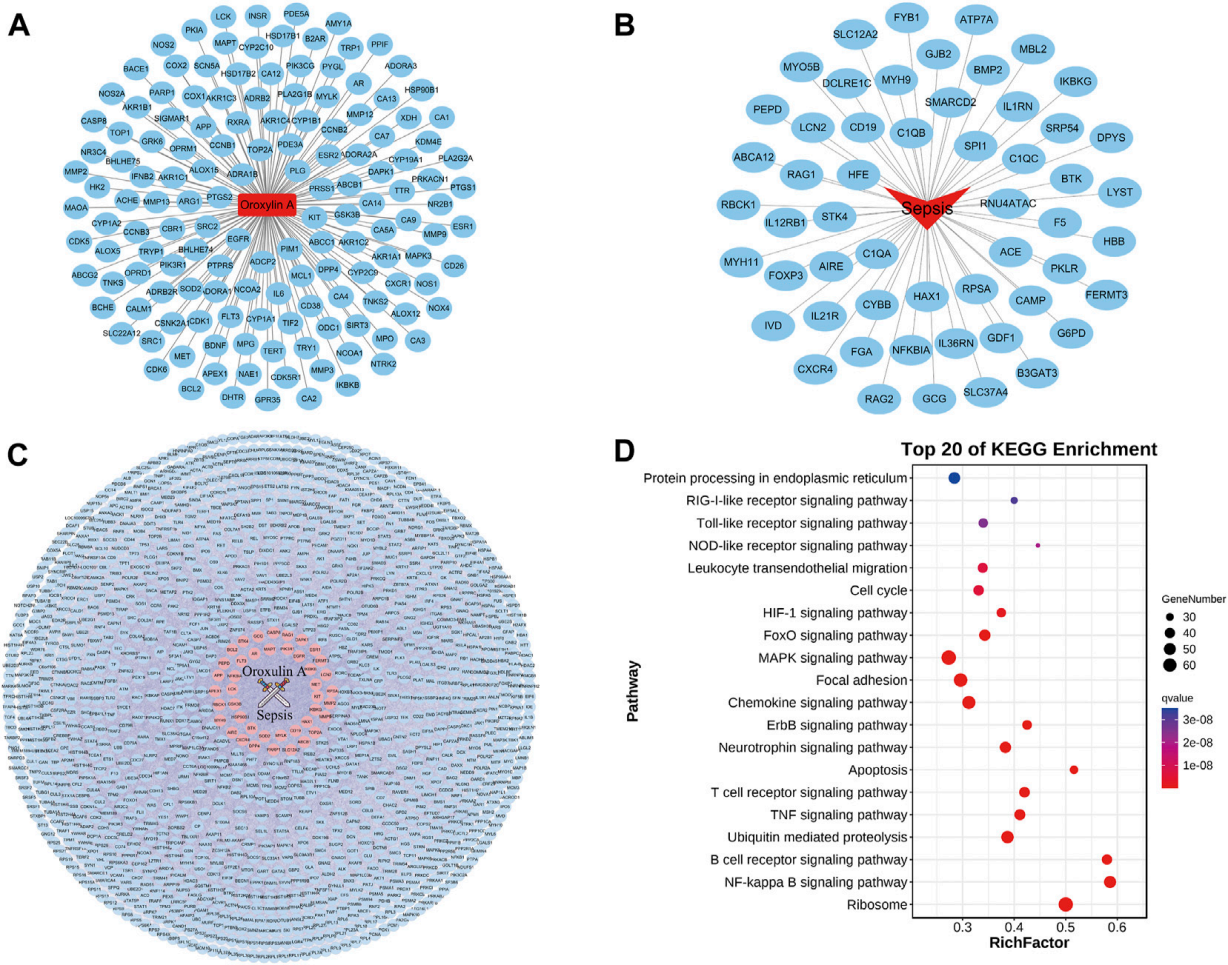
Pathway prediction of oroxylin A treating on sepsis. **(A)** Potential targets screen of oroxylin A. **(B)** Related targets screen of sepsis. **(C)** Intersection of candidate targets between oroxylin A and sepsis. **(D)** KEGG enrichment analysis of oroxylin A in the treatment of sepsis.

In order to explore the antisepsis mechanism of oroxylin A, network pharmacology was used to predict the pathways. A total of 147 targets that interacted with oroxylin A were screened from the PharmMapper server, SwissTargetPrediction database, and Drugbank (Figure 6A). A total of 54 targets related to sepsis were acquired from OMIM (Figure 6B). The intersection network of related targets PPI between oroxylin A and sepsis is shown in Figure 6C.

The KEGG enrichment results were revealed by a bubble graph. The number of covered genes of each pathway was represented by the size of the spot. The FDR (q value) indicated the significance of enrichment, of which the value was closer to 0, the more significant it was. The names of pathways on longitudinal coordinates were arranged according to the q values. The rich factor (horizontal coordinate) exhibited the enrichment index, which was proportional to the degree of enrichment. Taking all these factors, our results indicated that among the related biological pathways, the NF-κB signaling pathway might be important in the mechanism of oroxylin A on sepsis (Figure 6D).

#### 3.4.2 Verification of Pathway Using WB and SEAP Assay

**FIGURE 7:**
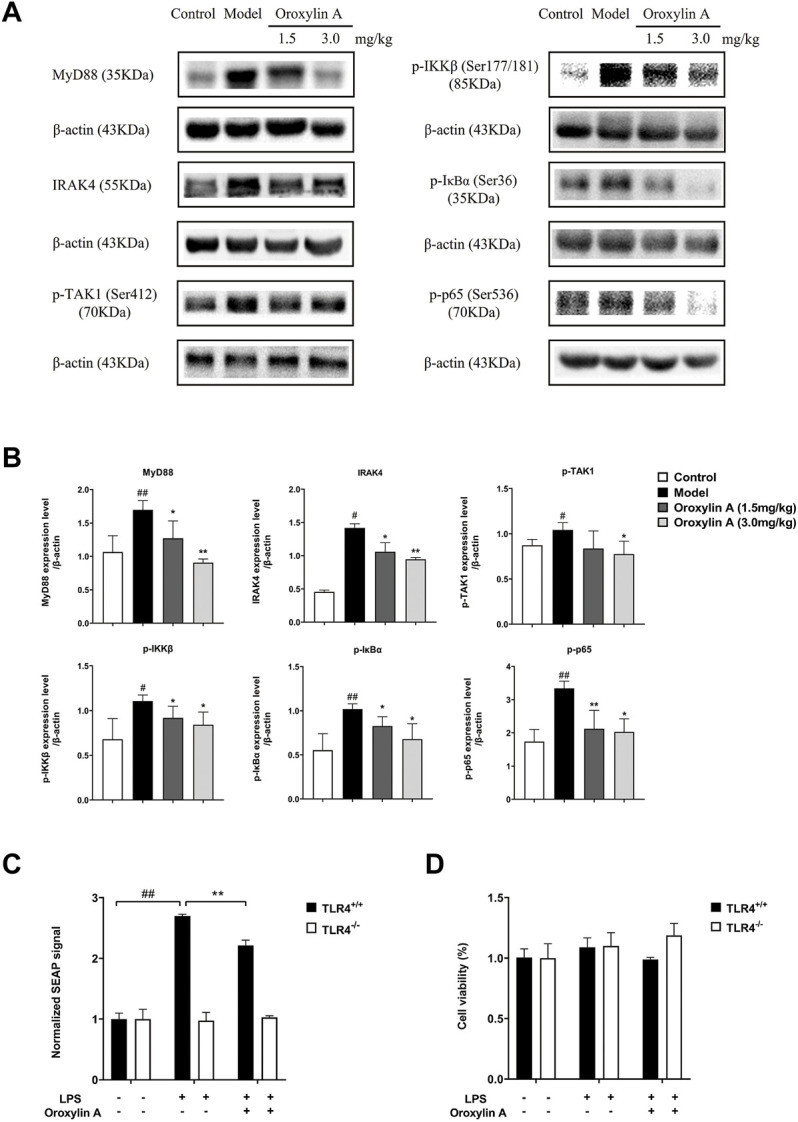
Oroxylin A suppressed the activation of TLR4/NF-κB signaling pathways *in vitro* and *in vivo*. **(A)** Related proteins of the TLR4-NFκB pathway evaluated by Western blot assay. **(B)** Statistical results of the protein levels of MyD88, IRAK4, p-TAK1, p-IKKβ, p-IκBα, and p-p65. The band intensity was normalized by β-actin and calculated according to the gray level. **(C)** Activities of oroxylin A (50 µM) in HEK-Blue hTLR4 cells and HEK-Blue null cells evaluated by measuring the expression of SEAP signal-induced by LPS (1 µg/ml). **(D)** Cell viability of HEK-Blue hTLR4 cells and HEK-Blue null cells with different treatment of LPS (1 µg/ml) or oroxylin A (50 µM) by MTT assay. The samples were *n* = 3 per group. Data are presented as mean ± SD. #*p* < 0.05 vs. control, ##*p* < 0.01 vs. control, *p < 0.05 vs. model, **p < 0.01 vs. model.

The spleen is an important lymphoid organ, which responds to blood-borne pathogens for the first time. Hence, the spleen protein was selected for the pathway study. TLR4 is a receptor for LPS, which activates the NF-κB signaling pathway through the cascade reaction to play a role in regulating inflammation. The related protein expression levels of the TLR4/NF-κB pathway were investigated by Western blotting. Compared to the control, the levels of MyD88, IRAK4, p-TAK1 (Ser412), p-IKKβ (Ser177/181), p-IκBα (Ser36), and p-p65 (Ser536) were upregulated in the LPS-induced model group. After being treated with oroxylin A, the aforementioned protein expression levels were significantly descended at 24 h after the LPS challenge (Figures 7A,B).

To further evaluate the effect of oroxylin A on the TLR4/NF-κB signaling pathway, the detection of NF-κB SEAP reporter in HEK-Blue hTLR4 cells was conducted. In the HEK-Blue hTLR4 cell, the expression of SEAP signal was significantly increased with the induction by LPS relative to the control (*p* < 0.0001). In contrast to the LPS-induced model group, the expression of SEAP signal was significantly reduced after being cultured by the oroxylin A-containing medium (*p* = 0.0008) (Figure 7C). In agreement with the results of WB, these results suggested that oroxylin A could interfere with the TLR4 signaling pathway and inhibit the transcription of NF-κB. Meanwhile, the SEAP signal was not activated by LPS in HEK-Blue null cells. The cell viability tested by MTT showed that there was no significant difference in cell activity among the three groups, which excluded the interference of cytotoxicity in the experiment (Figure 7D).

Combining the results of WB and SEAP, it can be seen that oroxylin A regulated the TLR4/NF-κB signaling pathway in endotoxemic mice.

## 4 Discussion

Among the plants of Lamiaceae family, only genus *Scutellaria* plants contain 4′-deoxyflavones (such as baicalein and wogonin). In addition, the 6 hydroxyl or 8 methoxy group on A-ring of the 4′-deoxyflavones has been found unusual in flavonoid compounds. As they are only synthesized in SR, they are called root-specific 4′-deoxyflavones. The root-specific 4′-deoxyflavones have anti-fibrosis, antivirus, and anticancer effects (Himeji et al., 2007; Huang et al., 2010; Fox et al., 2012; Yang et al., 2012; Nayak et al., 2014). More importantly, they have low or no toxicity to healthy cells (Baumann et al., 2008; Parajuli et al., 2009). In our previous studies, it was found that the anti-inflammatory activities of SR extracts from different ecological conditions varied wildly, which was mainly due to the dramatic difference in the chemical composition. According to this, SR was chosen as a small molecular library possessing abundant anti-inflammatory active compound, from which the potential antisepsis drugs were screened through the correlation analysis between plant metabolomics and pharmacodynamics. The components with a high correlation coefficient were further screened and verified by cellular and animal experiments. Finally, oroxylin A has been discovered, which can significantly improve the survival rate of endotoxemia mice both in LPS- and CLP-induced models. These results proved the feasibility of the screening strategy.

Oroxylin A has prominent anti-inflammatory activity, of which the mechanism has been considered to be related to the NF-κB pathway (Chen et al., 2000; Tseng et al., 2012; Yao et al., 2014). Consistent with that, our results of pathway prediction showed that oroxylin A might play an antisepsis through the NF-κB pathway. NF-κB is one of the key regulators of pro-inflammatory gene expression by binding to the NF-κB binding site in the promoter region of the target gene to induce the transcription of pro-inflammatory mediators (Lappas et al., 2002). The results of Elisa showed that oroxylin A significantly reduced the expression of pro-inflammatory cytokines in the plasma of endotoxemic mice, illustrating the regulation of NF-κB pathway. However, the mechanism of oroxylin A against sepsis is still ambiguous. In our results, the downstream proteins of TLR4 in the TLR4/NF-κB pathway were notably regulated by oroxylin A, suggesting the regulation of oroxylin A in the TLR4/NF-κB pathway. The detection results of NF-κB SEAP reporter in HEK-Blue hTLR4 cells further confirmed the previous conclusion. However, the results of SEAP also indicated that oroxylin A might not have strong TLR4 targeting specificity, suggesting that like most natural products, oroxylin A might play an antisepsis role through the multi-target and multi-pathway.

On the other hand, although oroxylin A did not show outstanding TLR4 targeting specificity, it could also provide useful information for the development of antisepsis drugs targeting TLR4. TLR4 and MD-2 on the cell surface synergistically induce the exogenous LPS. MD-2 and TLR4 jointly form the LPS-MD2-TLR4 complex on the cell membrane, resulting in the recruitment of adapter protein MyD88. Then, a series of downstream signals are activated and finally upregulate the expression of pro-inflammatory cytokines (Kumar et al., 2011; Moghimpour Bijani et al., 2012). It was reported that baicalein, a main component in SR, could directly bind to TLR4 to interrupt the formation of the complex (LPS-MD2-TLR4) (Chen et al., 2021). Luteolin, 3′,4′,5,7-tetrahydroxyflavone, was found to significantly improve the survival rate of endotoxemia mice by combining in an interface of LPS and MD-2 to prevent the formation of the complex (Yan et al., 2020). Hence, oroxylin A, with a similar structure to baicalein and luteolin, was speculated to have the similar binding mode directly with the TLR4 or LPS-MD2-TLR4 complex. Our molecular docking analysis indicated that oroxylin A may competitively inhibit LPS binding to MD-2 (Supplementary Figure S2A) *via* the hydrogen bonding with R90 and π–π interaction with F121 (Supplementary Figure S2B), which inhibited the formation of an activated dimer of TLR4 (Supplementary Figure S2C). After further confirmation of the binding sites, the structural optimization of oroxylin A can be carried out to obtain candidate drugs with better TLR4 specificity.

During the past decades, target-based drug discovery has been rapidly developed. The commonly used strategies were target-based chemical synthesis and compound library screen. Techniques like high-throughput screening (HTS), fragment-based screening, virtual screening, and computer-aided drug design (CADD) were used to find drugs with high affinity and selectivity for the target (Eder and Herrling, 2016). However, due to the complexity of biological systems, the compounds with superior efficacy at a protein level may have poor effects when come to the cellular and animal levels (Sams-Dodd, 2005). Compared to the target-based drug discovery, our cell-based screening strategy might have a greater chance of obtaining animal level effectiveness. In this study, the ecological environment was taken as a disturbance factor. Through the correlation analysis between the component content changes of SR and the corresponding anti-inflammatory activity, the components with outstanding anti-inflammatory activity were quickly screened out, which was time-saving and of low cost.

Because biological networks are redundant at various levels, the one-target one-drug paradigm has been found not applicable to all diseases, such as polygenic diseases and viral infections. In order to overcome the limitations of single drug target, designing drugs that can act on multiple targets simultaneously and specifically has become a new direction of drug research (Yang et al., 2008; Yoon, 2011; Luo et al., 2019). High-content screening (HCS) technology, a cell-based type of phenotypic screening, has played an important role in the development of multi-target drugs (Fraietta and Gasparri, 2016; Sidarovich et al., 2018). Compared with HCS, the compound library used in our study was the extract of SR rich in anti-inflammatory active compounds, of which the hit-ratio could be higher. Nevertheless, due to the lack of enough reference standards, some compounds with high correlation coefficients have not been accurately identified, which might result in the missing of potential antisepsis components with superior activity.

In this study, SR was used as a natural small molecule library, and a screening strategy for potential antisepsis drugs was established based on the correlation analysis between plant metabolomics and anti-inflammatory activity. Furthermore, the antisepsis mechanism of oroxylin A was explored. Although the number of candidate compounds for accurate structural identification was limited, this might lead to the missing compounds with better efficacy. The study provided a time-saving method with a higher hit-ratio for screening antisepsis candidate drugs from TCM. These findings suggested that oroxylin A might be used as a platform for the design and synthesis of small molecule drugs for sepsis. Also, it also can be used as a chemical probe to find more target information for clinical treatment and drug discovery of sepsis. From the foregoing, our results provided a feasible screening strategy and data support for the development of antisepsis drugs.

## Data Availability

The original contributions presented in the study are included in the article/Supplementary Material, further inquiries can be directed to the corresponding authors.
