# Supplementary material for: A Candidate Drug Screen Strategy: The Discovery of Oroxylin A in Scutellariae Radix Against Sepsis via the Correlation Analysis Between Plant Metabolomics and Pharmacodynamics
Source: Front Pharmacol. 2022 May 19;13:861105. doi: 10.3389/fphar.2022.861105 (PMC9160923; doi:10.3389/fphar.2022.861105)
Supplement: Supplementary file 1 [file DataSheet1.docx]

Supplementary Material

# Supplementary Figures and Tables

## Supplementary Tables

**Table S1** Information of different ecological Scutellariae Radix samples

| No. | Producing areas | Growth years | Harvest season |
| --- | --- | --- | --- |
| 1 | Nianzhang Town, Xia County, Yuncheng City, Shanxi Province | 1.5 years | Autumn |
| 2 | Nianzhang Town, Xia County, Yuncheng City, Shanxi Province | 2.5 years | Autumn |
| 3 | Nianzhang Town, Xia County, Yuncheng City, Shanxi Province | 3.5 years | Autumn |
| 4 | Miaoqian Town, Xia County, Yuncheng City, Shanxi Province | 1.5 years | Autumn |
| 5 | Miaoqian Town, Xia County, Yuncheng City, Shanxi Province | 2.5 years | Autumn |
| 6 | Miaoqian Town, Xia County, Yuncheng City, Shanxi Province | 4.5 years | Autumn |
| 7 | Longxi County, Dingxi City, Gansu Province | 2 years | Autumn |
| 8 | Yuncheng City, Shanxi Province | 2 years | Autumn |
| 9 | Wenshui Town, Pingyi County, Shandong Province | 2 years | Autumn |
| 10 | Shagou Town, Yishui County, Shandong Province | 1 year | Autumn |
| 11 | Anguo City, Hebei Province | 1.5 years | Autumn |
| 12 | Heyang County, Shaanxi Province | 2 years | Autumn |
| 13 | Chengde City, Hebei Province | 4-5 years  (Imitative wild) | Autumn |
| 14 | Chengde City, Hebei Province | 4-5 years  (Imitative wild) | Spring |
| 15 | Wanrong County, Yuncheng City, Shanxi Province | 2 years and 4 months | Autumn |
| 16 | Wanrong County, Yuncheng City, Shanxi Province | 2.5 years | Spring |
| 17 | Xia County, Yuncheng City, Shanxi Province | 2 years and 4 months | Autumn |
| 18 | Xia County, Yuncheng City, Shanxi Province | 2.5 years | Spring |
| 19 | Longxi County, Dingxi City, Gansu Province | 2 years and 7 months | Autumn |
| 20 | Longxi County, Dingxi City, Gansu Province | 3 years | Spring |

**Table S2** Components with score ≥ 80 in correlation analysis of plant metabolomics and pharmacodynamics

| No. | Identification | Correlation coefficient  (negative mode) | Correlation coefficient  (positive mode) |
| --- | --- | --- | --- |
| 1 | Chrysin | 96.7 | 96.5 |
| 2 | Wogonin | 92.7 | 94.9 |
| 3 | Pinocembrin | 95.8 | 94.5 |
| 4 | Baicalein | 95.9 | 92.9 |
| 5 | Oroxylin A | 88.4 | 90.5 |
| 6 | Baicalein-7-O-β-D-glucoside | 90.2 | 89.7 |
| 7 | Acteoside | 91.6 | 89.3 |
| 8 | Isoacteoside | <80 | 85.9 |
| 9 | 8,8''-Bibaicalein | <80 | 87.4 |
| 10 | Scutellarin | 81.2 | <80 |

## Supplementary Figures


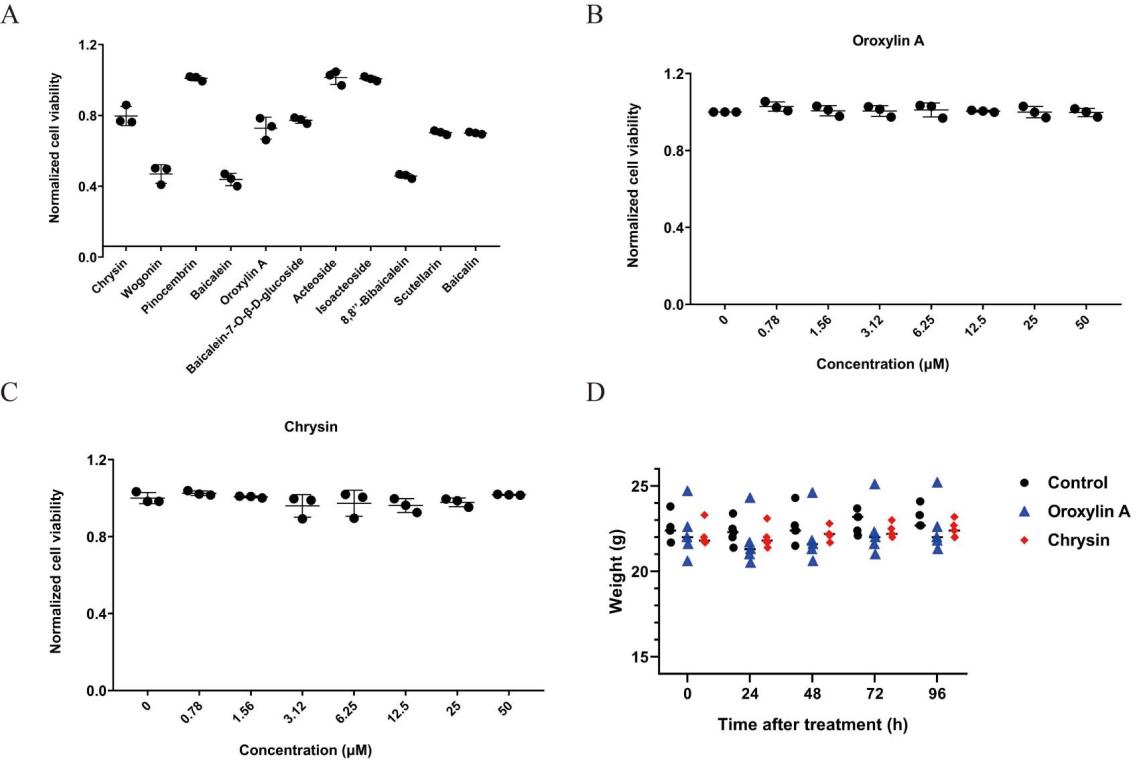


**Supplementary Figure 1.** Toxicity of compounds to cells and mice at the experimental dose. (A) Cells were treated with 100 μM corresponding reagents and then measured by MTT assay, following by the normalization of cell viability. Cells were treated with serial concentration of Oroxylin A (B) or Chrysin (C) (0.78, 1.56, 3.12, 6.25, 12.5, 25 and 50 µM) and then measured by MTT assay, following by the normalization of cell viability. All data were reported as the mean of three independent experiments. (D) Effect of Oroxylin A and Chrysin on the weight of mice (n = 5). Compounds (3.0 mg/kg) were inject to mice every day


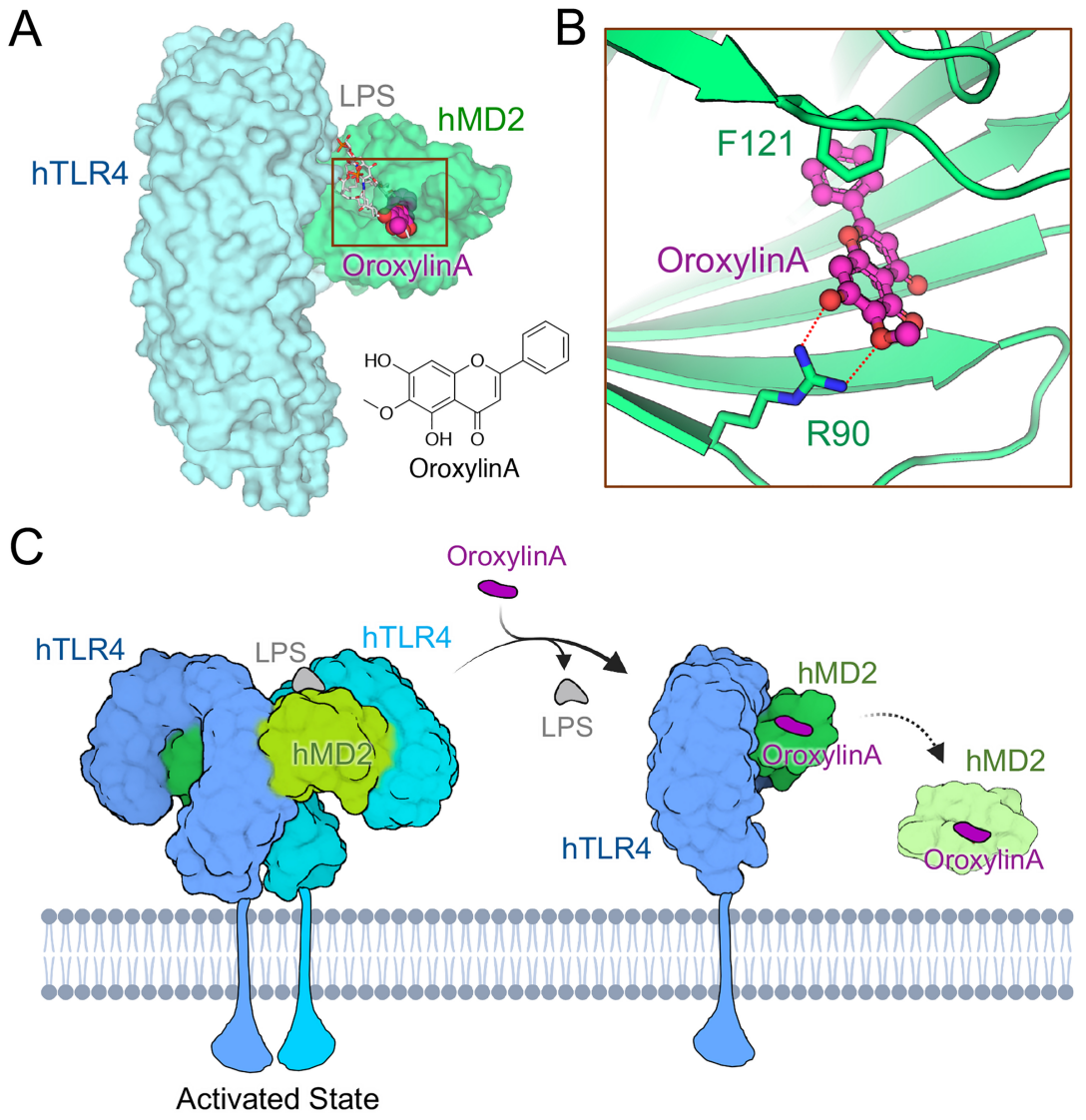


**Supplementary Figure 2.** Oroxylin A might competitively inhibit LPS binding to MD-2. (A) Overview of oroxylin A docked to hMD-2. (B) Close-up view of interactions between oroxylin A and hMD-2. (C) Schematic representation of TLR4 activation failure due to competitive binding of oroxylin A to MD-2.
